# Supplementary material for: Human bladder organoids model urinary tract infection and bacteriophage therapy
Source: bioRxiv. 2025 Jul 30:2025.07.30.667685. Preprint. [Version 1] doi: 10.1101/2025.07.30.667685 (PMC12324606; doi:10.1101/2025.07.30.667685)
Supplement: Supplement 1 [file media-1.pdf]

SUPPLEMENTAL MATERIALS

Human bladder organoids model urinary tract infection and bacteriophage therapy

Jacob J. Zulk, Clare M. Robertson, Samantha Ottinger, Amal Kambal, Alejandra Rivera Tostado, Rachel C. Fleck, Allyson E. Shea, Cristian Coarfa, Sarah E. Blutt, Anthony W. Maresso, Kathryn A. Patras

Contents:

Supplemental Figures 1-2

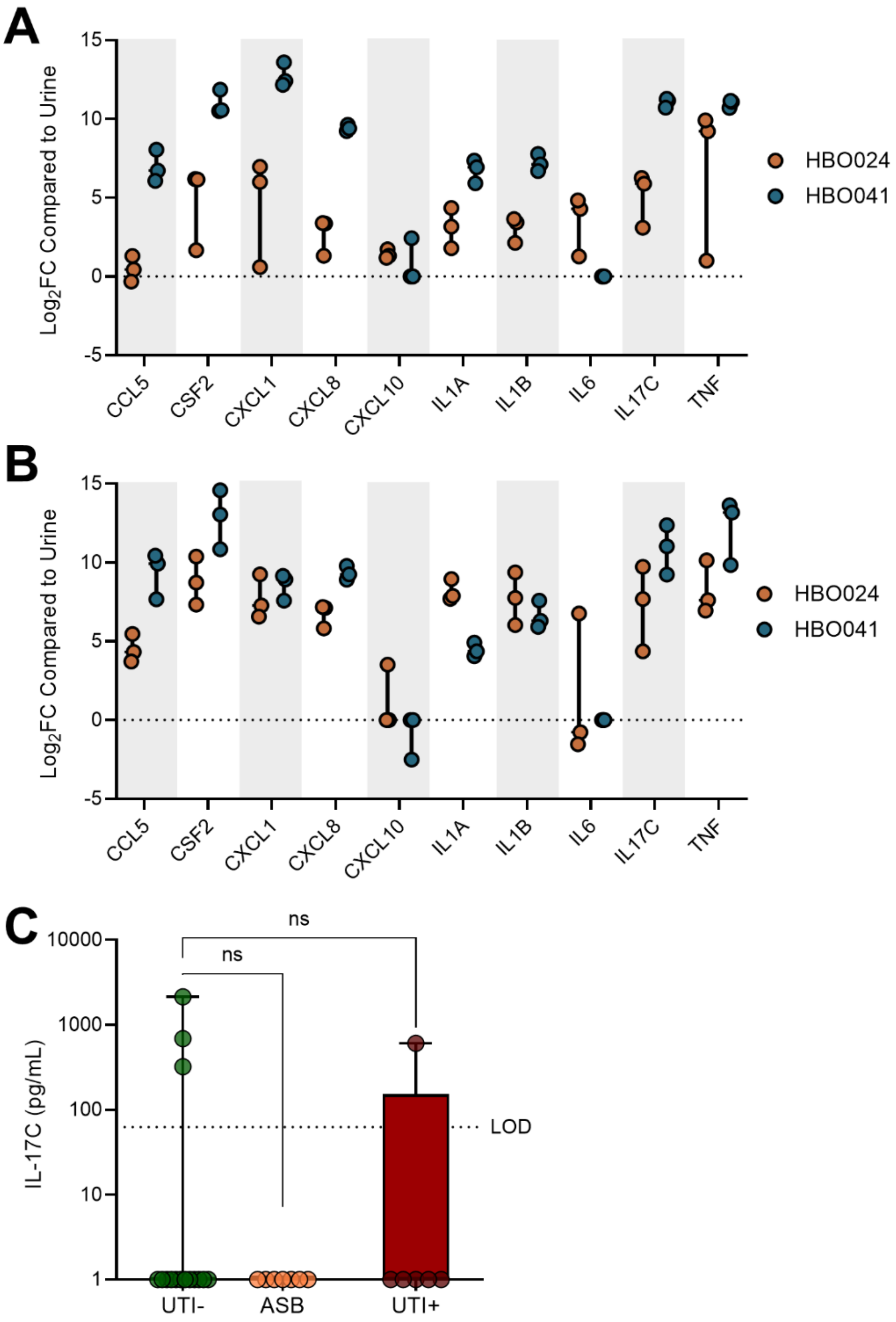

Supplementary Figure 1: Comparison of relative cytokine expression between the two lines following UPEC infection. Log_2 fold-change in inflammatory-related cytokines compared to urine control for both organoid lines after 4 (A) and 24 (B) hours of infection. This data is the expanded version of the heatmap shown in **Fig. 3E**. (C) IL-17C quantification from the urine of women with UTI, asymptomatic bacteriuria (ASB), or healthy controls. Data were analyzed using Kruskal-Wallis test with Dunn's multiple comparisons (C).

A

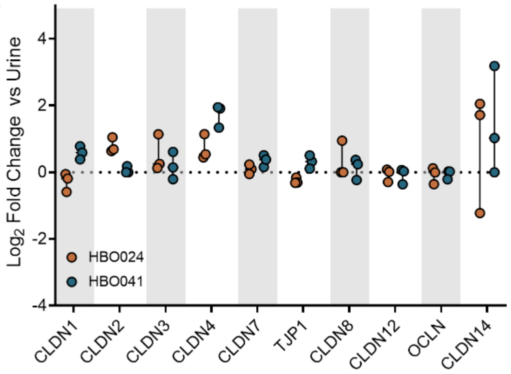

B

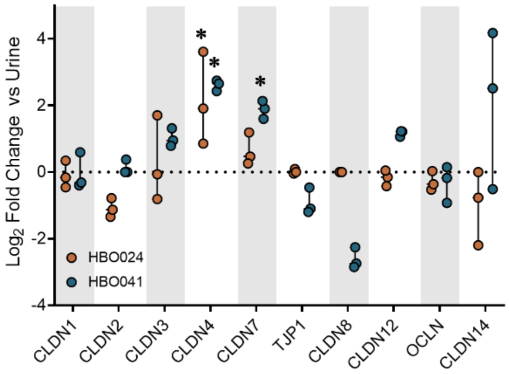

C

	HBO024 4h			HBO041 4h			HBO024 24h			HBO041 24h		
Gene	Log2FC	pvalue	padj	Log2FC	pvalue	padj	Log2FC	pvalue	padj	Log2FC	pvalue	padj
CLDN1	-0.2376949	1	1	0.55922082	1	1	-0.1070513	1	1	-0.0765988	1	1
CLDN2	0.87436107	1	1	-0.2241595	1	1	-1.0543127	1	1	-0.0393984	1	1
CLDN3	0.45725976	1	1	0.12745249	1	1	0.43268962	1	1	0.99844276	1	1
CLDN4	-0.0442139	1	1	1.69198336	0.06299358	1	2.39128321	0.00018538	0.00933724	2.61203607	1.48E-05	0.00029652
CLDN7	0.08490005	1	1	0.309171	1	1	0.65289837	1	1	1.91104044	0.00114655	0.01302329
CLDN8	0.73780259	1	1	0.22370675	1	1	0	1	1	-2.6207435	0.04939415	0.26669336
CLDN12	-0.0803435	1	1	-0.0826325	1	1	-0.1566982	1	1	1.16991179	0.40310696	1
CLDN14	1.57213326	0.81781232	1	4.32342076	0.19875585	1	-2.2387161	0.63443286	NA	3.09548257	0.36814108	NA
ZO-1 (TJP1)	-0.2519829	1	1	0.32084132	1	1	0.01596271	1	1	-0.947094	1	1
Occludin (OCLN)	-0.0499858	1	1	-0.0334613	1	1	-0.2690963	1	1	-0.2590782	1	1

D

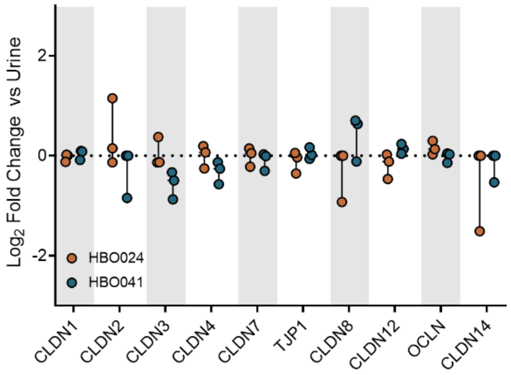

E

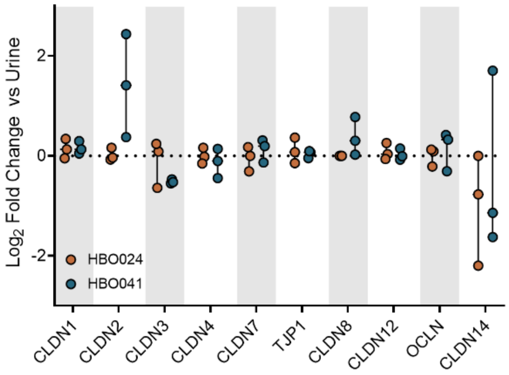

F

	HBO024 4h			HBO041 4h			HBO024 24h			HBO041 24h		
Gene	Log2FC	pvalue	padj	Log2FC	pvalue	padj	Log2FC	pvalue	padj	Log2FC	pvalue	padj
CLDN1	-0.0284326	1	1	0.04743559	1	1	0.14210098	1	1	0.16593575	1	1
CLDN2	0.25338701	1	1	-2.180711	0.47764088	1	0.02325383	1	1	3.33246132	0.09030864	1
CLDN3	-0.0029352	1	1	-0.4825235	1	1	-0.122093	1	1	-0.5174662	1	1
CLDN4	-0.0179751	1	1	-0.272392	1	1	-0.0156606	1	1	-0.1035021	1	1
CLDN7	-0.0280815	1	1	-0.1324321	1	1	-0.0462708	1	1	0.11820304	1	1
CLDN8	-1.8856951	0.61467157	1	0.39751798	1	1	0	1	1	0.32974321	1	1
CLDN12	-0.1997877	1	1	0.14022655	1	1	0.08044955	1	1	0.03538591	1	1
CLDN14	-2.3878092	0.60720594	1	0.61089941	1	1	-3.6525448	0.30857678	1	-0.5115528	1	1
ZO-1 (TJP1)	-0.1211667	1	1	0.01706251	1	1	0.11674433	1	1	0.03745133	1	1
Occludin (OCLN)	0.14058459	1	1	-0.005417	1	1	0.00332819	1	1	0.17049534	1	1

Supplementary Figure 2: Regulation of HBO tight junction proteins following UPEC infection and HP3 treatment. Log2 fold-change in expression of bladder tight junction proteins following UPEC infection after (A) 4 and (B) 24 hours of UPEC infection, relative to urine only treated HBOs. (C) Table showing fold-change and p-values for bladder tight junction protein expression following UPEC infection. Log2 fold-change in expression of bladder tight junction proteins following HP3 treatment after (D) 4 and (E) 24 hours of incubation, relative to urine only treated HBOs. (F) Table showing fold-change and p-values for bladder tight junction protein expression following HP3 treatment.
